# Relationship between Abdominal Pressure, Pulmonary Compliance, and Cardiac Preload in a Porcine Model

**DOI:** 10.1155/2012/763181

**Published:** 2012-02-20

**Authors:** Joost Wauters, Piet Claus, Nathalie Brosens, Myles McLaughlin, Greet Hermans, Manu Malbrain, Alexander Wilmer

**Affiliations:** ^1^Medical Intensive Care Unit, University Hospital Gasthuisberg, Herestraat 49, 3000 Leuven, Belgium; ^2^Cardiac Imaging, University Hospital Gasthuisberg, 3000 Leuven, Belgium; ^3^Intensive Care Unit, Ziekenhuis Netwerk Antwerpen, Campus Stuivenberg, 2060 Antwerpen, Belgium

## Abstract

*Rationale*. Elevated intra-abdominal pressure (IAP) may compromise respiratory and cardiovascular function by abdomino-thoracic pressure transmission. We aimed (1) to study the effects of elevated IAP on pleural pressure, (2) to understand the implications for lung and chest wall compliances and (3) to determine whether volumetric filling parameters may be more accurate than classical pressure-based filling pressures for preload assessment in the setting of elevated IAP. *Methods*. In eleven pigs, IAP was increased stepwise from 6 to 30 mmHg. Hemodynamic, esophageal, and pulmonary pressures were recorded. *Results*. 17% (end-expiratory) to 62% (end-inspiratory) of elevated IAP was transmitted to the thoracic compartment. Respiratory system compliance decreased significantly with elevated IAP and chest wall compliance decreased. Central venous and pulmonary wedge pressure increased with increasing IAP and correlated inversely (*r* = −0.31) with stroke index (SI). Global end-diastolic volume index was unaffected by IAP and correlated best with SI (*r* = 0.52). *Conclusions*. Increased IAP is transferred to the thoracic compartment and results in a decreased respiratory system compliance due to decreased chest wall compliance. Volumetric filling parameters and transmural filling pressures are clearly superior to classical cardiac filling pressures in the assessment of cardiac preload during elevated IAP.

## 1. Introduction

Elevated intra-abdominal pressure (IAP) is commonly encountered in critically ill patients as a result of abdominal diseases or conditions associated with bowel distention, ascites, peritonitis, hemoperitoneum, or trauma [1]. Based on recent consensus definitions, intra-abdominal hypertension (IAH) is diagnosed as a consistently increased IAP value of at least 12 mmHg [2, 3]. Previous studies demonstrated negative effects of elevated IAP on respiratory function: a reduction in respiratory system compliance and increased airway pressures through elevation of the diaphragm [4–7]. In order to differentiate between the contribution of lung and chest wall in decreased respiratory system compliance, pleural pressure must be measured. The first aim of the present study was to analyze the effects of elevated IAP on pleural pressures at different stages of the respiratory cycle and the implications for measurements of lung or chest wall compliance and abdomino-thoracic pressure transmission.

Cardiovascular consequences of IAH include a reduction in cardiac output and stroke volume, resulting from both a decreased venous return and an increase in systemic vascular resistance [4, 8]. Since central venous pressure (CVP) and pulmonary artery occlusion pressure (PAOP) typically increase with rising IAP in combination with decreasing stroke volume, determination of filling status based on CVP and PAOP is difficult in IAH [9–11]. Therefore, a second aim was to study the effect of elevated IAP on different clinical markers of preload status: pressure-based parameters (CVP, PAOP), transmural pressure-based parameters incorporating a correction for pleural pressure and volumetric parameters. This study was conducted in a porcine model of elevated IAP.

## 2. Methods

### 2.1. Animal Instrumentation

This study was performed in accordance with the guidelines and after explicit approval of the local Institutional Ethics Committee on Animal Care and Use. After overnight fasting with free access to water, 11 domestic pigs (mean body weight of 45 ± 5 kg) were anaesthetized with 4 mg/kg of tiletamine and 4 mg/kg zolazepam (Zoletil, Virbac, Barneveld, The Netherlands), in combination with 3 mg/kg xylazine (Xyl-M, VMD, Breendonk, Belgium), all intramuscularly. A polyethylene catheter with a thin-walled, flaccid latex balloon (10 cm long) sealed over one end of the catheter (International Medical, Zutphen, The Netherlands) was passed through the snout into the oesophagus, the pigs being in supine position without restraints. The catheter was first positioned into the stomach. This position was confirmed by an increase in the balloon pressure during a spontaneous inspiratory effort. The catheter was then withdrawn to the point where pressure swings reversed direction resulting in negative inspiratory pressures, indicating that the balloon had entered the thoracic cavity. Finally, the catheter was withdrawn another 10 cm and was fixed to the snout. Pigs were intubated and mechanically ventilated (Servo 900C, Siemens) using an inspiratory oxygen concentration (fiO2) of 0.5, a constant tidal volume (TV) of 9 mL/kg, an inspiration/expiration ratio of 1 : 2 and a positive end-expiratory pressure (PEEP) of 5 cmH2O. The respiratory rate was adjusted to maintain arterial pCO_2_ between 35–45 mmHg. Anaesthesia was maintained by continuous infusion of 7 mg*·*kg^−1^ *·*h^−1^ of propofol (Diprivan, AstraZeneca, Brussels, Belgium), 0.1 mg*·*kg^−1^ *·*h^−1^ pancuronium (Pavulon, Organon, The Netherlands) and with a bolus of 0.25 mg*·*kg^−1^ buprenorphine (Temgesic, Schering-Plough, Brussels, Belgium) every 3 h. A 5 F thermistor-tipped catheter (Pulsion Medical Systems, Munich, Germany) was placed in the descending aorta via the left femoral artery. Via a midneck incision, a pulmonary artery thermodilution catheter (Baxter Healthcare Corp., Irvine, CA, USA) was placed in the pulmonary artery via the right external jugular vein. Hartmann/Ringer lactate at 4 mL·kg^−1^·h^−1^ (Viaflex, Baxter, Lessines, Belgium), glucose 50% at 0.1 g*·*kg^−1^ *·*h^−1^, and HAES-steril at 5 mL·kg^−1^·h^−1^ (FreeFlex, Fresenius, Friedberg, Germany) were administered as continuous infusion together with anaesthetic drugs via an additional 3-lumen catheter in the left external jugular vein. Core temperature was maintained at 38.5 ± 0.5°C using a heating blanket. A midline laparotomy was performed. An air-capsule pressure catheter (Spiegelberg, Hamburg, Germany) was placed in the right lower abdomen, and a drain was placed in the left lower abdomen for intraperitoneal saline infusion. A catheter was inserted into the bladder by a transdermal suprapubic puncture for urine collection. All catheters were exteriorised and the laparotomy was carefully closed in two layers.

### 2.2. Measurements and Calculations: Pulmonary Mechanics and Pulmonary Function

Peak inspiratory pressure (PIP) and end-inspiratory plateau pressure (Pplat) were recorded from the ventilator. Esophageal pressure (Peso), as a surrogate for pleural pressure, was determined end-inspiratory (eiPeso) and end-expiratory (eePeso) by inflating the balloon with 0.75 mL of air. Total compliance of the respiratory system (Crs) was calculated as TV/(Pplat−PEEP). Chest wall compliance (Ccw) was calculated as TV∗0.75/(eiPeso−eePeso) and lung compliance (Clung) as TV/((Pplat−PEEP)−((eiPeso−eePeso)/0.75)). Because airway pressures are expressed in cmH2O and esophageal pressures in mmHg, we used 0.75 to convert units when necessary. Blood samples were taken from the left femoral artery and arterial blood gas values (paO2, paCO2) were determined in a blood gas analyser (ABL System 625, Radiometrics Medical, Copenhagen, Denmark).

### 2.3. Measurements and Calculations: Global Hemodynamics

Mean arterial pressure (MAP), heart rate (HR), central venous pressure (CVP), mean pulmonary artery pressure (MPAP), and end-expiratory pulmonary artery occlusion pressure (PAOP) were measured. All pressures were zeroed at the midchest level. No vasoactive drugs were administered. To assess filling pressures in the context of elevated IAP, we calculated transmural mean CVP (tCVP) as CVP−mPeso, with mPeso being calculated as ((2∗eePeso + eiPeso)/3). Transmural end-expiratory pulmonary artery occlusion pressure (tPAOP) as POAP−eePeso. After injecting a bolus of 15 mL of iced saline into the superior vena cava, stroke index (SI) was calculated from cardiac index (CI) as CI/HR. CI was determined from transpulmonary thermal indicator dilution at the femoral artery. Systemic vascular resistance (SVRI) was calculated as (MAP−CVP)/CI. Based on the mean transit time and the exponential downslope time of the thermodilution curve, global end-diastolic volume index (GEDVI) and extravascular lung water index (EVLWI) were calculated with the PICCO system [12–14]. SI and intrathoracic blood volume are reported as the average of three repeated measurements lying within 10% range. Indices are calculated based on bodyweight. Intra-abdominal pressure (IAP) was measured with an IAP monitor (Spiegelberg, Hamburg, Germany) [15].

### 2.4. Experimental Protocol

After a stabilization period of 2 hours following surgical preparation, a baseline measurement was made and respiratory settings and infusions rates were kept constant during the rest of the protocol. Then IAP was increased sequentially to 10, 20, and 30 mmHg by infusing warmed (38°C) saline into the peritoneal cavity. The animals were maintained at each IAP level for 45 minutes and then measurements were obtained. After the measurement at 30 mmHg, saline was drained. 45 minutes later, the last dataset was acquired and animals were sacrificed by hypertonic potassium chloride injection under deep anaesthesia.

### 2.5. Statistical Analysis

Results are derived from one measurement per IAP level per pig and are expressed as mean ± SEM. Since data were not normally distributed, a Friedman analysis of variance was used to analyze the evolution of parameters over different IAP levels. In case of significant results, a post hoc multiple comparison analysis was performed using a Bonferroni correction. For correlations, Spearman coefficient of correlation (*r*) was calculated (Statistica 7.0, StatSoft Inc., Tulsa, USA). For all tests, *P* ≤ 0.05 was considered statistically significant.

## 3. Results

### 3.1. Effects of Elevated IAP on Abdominothoracic Pressure Transmission, Pulmonary Compliance, and Pulmonary Function

**Figure 1:**
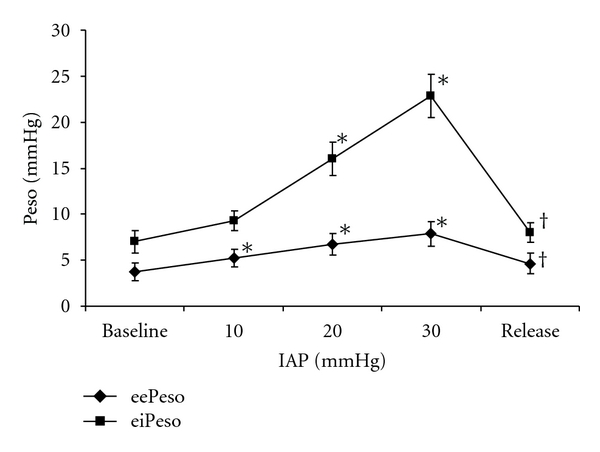
Effect of increasing intra-abdominal pressure (IAP) on end-expiratory esophageal pressure (eePeso) and end-inspiratory esophageal pressure (eiPeso). Baseline = 6 mmHg, release = 5 mmHg. Data are expressed as mean ± SEM. *P = 0.04 versus baseline, ^†^ *P* = 0.04 versus IAP = 30.

**Figure 2:**
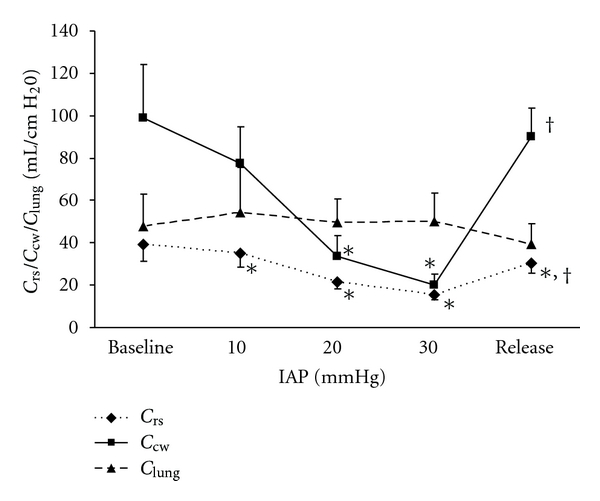
Effect of increasing intra-abdominal pressure (IAP) on respiratory system compliance (Crs), chest wall compliance (Ccw), and lung compliance (Clung). Baseline = 6 mmHg, release = 5 mmHg. Data are expressed as mean ± SEM. *P = 0.04 versus baseline, ^†^ *P* = 0.04 versus IAP = 30.

IAP was 6 ± 1 mmHg before (baseline) and 5 ± 1 mmHg at the end of the experiment (release). Both, eePeso and eiPeso increased significantly with increasing IAP. The increase in eiPeso was much more pronounced (Figure 1). After abdominal decompression, eePeso and eiPeso returned to baseline values. Using eePeso, 17% of IAP was transmitted from the abdominal to the thoracic compartment as shown by linear regression with eePeso = 0.17∗IAP + 4.2 (*r* = 0.6, *P* = 0.00001). Using eiPeso or mPeso, transmission was 62% and 33%, respectively, (*P* = 0.000001). With increasing IAP, both Crs and Ccw decreased significantly (Figure 2). The decrease was more pronounced for Ccw and showed a strong inverse correlation with IAP (*r* = −0.84, *P* = 0.00001). Clung did not change significantly over the whole IAP range. After decompression, Ccw returned to baseline but Crs remained significantly lower than baseline. 

Other effects of increasing IAP on pulmonary dynamics and function are summarised in Table 1. As expected, Pplat and PIP increased significantly with increasing IAP, with Pplat being 0.52∗IAP + 8 (*r* = 0.87, *P* = 0.000001). PaCO2, PaO2/fiO2, and EVLWI were not significantly affected by increasing IAP.

### 3.2. Effects of IAP on Global Hemodynamics

**Figure 3:**
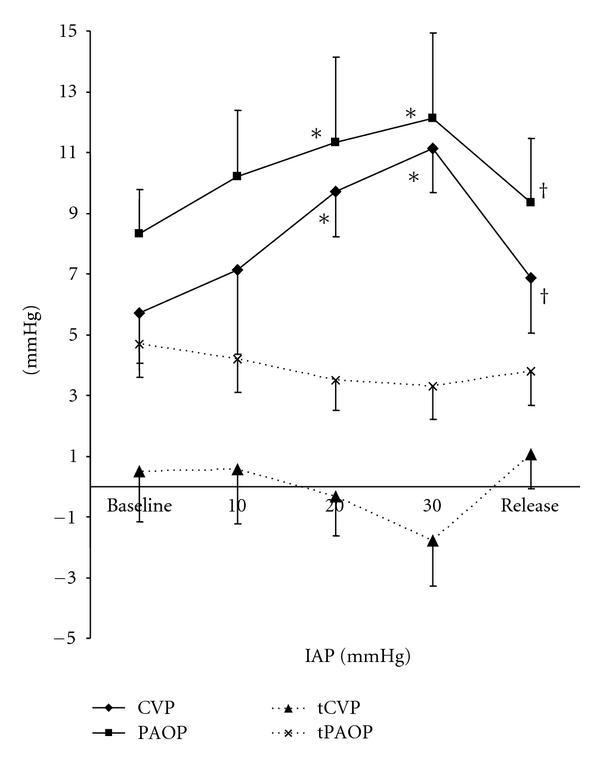
Effect of increasing intra-abdominal pressure (IAP) on central venous pressure (CVP), end-expiratory pulmonary artery occlusion pressure (PAOP) and transmural filling pressures (tCVP and tPAOP). Baseline = 6 mmHg, release = 5 mmHg. Data are expressed as mean ± SEM. *P = 0.04 versus baseline, ^†^ *P* = 0.05 versus IAP = 30.

While heart rate (HR) remained constant over the whole IAP range, SI decreased with increasing IAP (Table 1). Simultaneously, mean systemic and pulmonary pressures increased significantly in parallel with a significant rise in SVRI. After IAP release, MAP and SVRI showed a decrease towards baseline level, although MPAP stayed 30% above baseline value (Table 1). Filling pressures like CVP and PAOP also increased significantly in response to increasing IAP (Figure 3), with CVP = 0.22∗IAP + 5.6 (*r* = 0.65, *P* = 0.000001) and PAOP = 0.18∗IAP + 6.9 (*r* = 0.49, *P* = 0.001). CVP and PAOP both returned nearly to baseline values after decompression. Transmural filling pressures (tCVP and tPAOP) showed a decreasing trend with increasing IAP (Figure 3). While PAOP and CVP correlated inversely with SI (*r* = −0.31, *P* = 0.02), tCVP and tPAOP showed a moderate positive correlation with SI (*r* = 0.36, *P* = 0.03). GEDVI, as a volumetric preload estimator, remained quite constant over the different levels of IAP but correlated better with SI (*r* = 0.52, *P* = 0.04) than transmural filling pressures did.

## 4. Discussion

The main findings of this study are, first, that measurement of transfer of IAP to the thoracic compartment is dependent on the stage of the respiratory cycle in which pleural pressures are measured. Second, IAH results in a decreased respiratory system compliance due to a steep decrease of chest wall compliance. Third, transmural filling pressures and GEDVI are clearly superior to classical cardiac filling pressures such as CVP or PAOP in the assessment of cardiac preload at high intra-abdominal pressures.

### 4.1. Effects of IAP on Abdominothoracic Pressure Transmission, Pulmonary Compliance, and Pulmonary Function

We observed a transmission of IAP to the thoracic compartment of 17%, 33%, and 62%, when using *end-expiratory*, *mean,* or *end-inspiratory* pleural pressure, respectively. This is in agreement with others reporting IAP transmissions of 35–60% using *mean* pleural pressure and 55–70% based on *end-inspiratory* pleural pressure assessment [9, 16–18]. Quintel et al. described no effect on *end-expiratory* pleural pressure in pigs, but IAP was increased only to 15 mmHg [19]. Although some controversy exists on the accuracy of Peso measurement, several authors found Peso to be a good estimator of pleural pressure in the middle lung [20–23]. Moreover, the fact that we found similar fractions of IAP transmission to intrathoracic vascular pressures (22% and 18% of IAP was transmitted to CVP and end-expiratory PAOP, resp.) and ventilator pressures (52% of IAP was transmitted to Pplat) as to Peso independently confirms our findings on Peso data.

We observed reduced Crs with increasing IAP. This reduction in Crs was largely attributable to a decrease in Ccw. These findings are in agreement with others, showing similar decreases in Ccw during IAH, due to elevation of the diaphragm, increasing diaphragmatical stiffness and pleural effusion [6, 18, 19, 24, 25]. Although EVLWI did not change, suggesting the absence of important alveolar edema, Clung and paO2 tend to decrease at high IAP and after decompression, indeed suggesting initial degradation of pulmonary function. Data from others clearly show that IAH results in pulmonary dysfunction due to reduction of lung volumes, formation of atelectasis and promotion of lung edema [26]. In this study, the duration of applied IAH is probably too short to observe these effects on pulmonary function and this is one of the main limitations of this study.

### 4.2. Effects of IAP on Global Hemodynamics

Heart rate (HR) remained constant throughout the experiment, probably due to effects of anesthesia. SI showed an initial increase (8%) at 10 mmHg of IAP, followed by a decrease (22%) at 30 mmHg of IAP. In agreement with others, we hypothesize that this evolution in SI resulted from an initial autotransfusion effect from the abdominal compartment, followed by a decrease in venous return in combination with an increased afterload at high IAP [27]. Filling status clearly influences the relationship between IAP and SI. Kashtan et al. reported a decrease in CO of 53% in hypovolemic and 17% in normovolemic dogs when increasing IAP to 40 mmHg by abdominal fluid infusion [8]. Moreover, these authors showed that intravascular volume expansion (with approximately one third of the original intravascular volume) restored CO to normal during IAH [9, 28].

The importance of accurate preload assessment in managing cardiovascular dysfunction in IAH raises concern about the reliability of pressure-based preload assessment in patients with IAH. Elevated intrathoracic pressures, as a result of transdiaphragmatic IAP transmission, have been demonstrated to erroneously increase PAOP and CVP, with low CO [9, 10]. Our data have confirmed this by showing a strong correlation between Peso and CVP (*r* = 0.75) or PAOP (*r* = 0.7). We even observed weak but significant negative correlations between CVP or PAOP and SI, making CVP and PAOP not useful as preload estimators. Our findings are in good agreement with others demonstrating a moderate negative correlation between CVP and CO in a porcine model of long-term IAH (30 mmHg) [29–31].

Some authors advocated the use of transmural filling pressures to assess preload in the setting of IAH or high PEEP [9, 20, 31]. By using Peso to estimate pericardial pressure, we found both tCVP and tPAOP to decrease when IAP was increased. In addition, we could demonstrate tCVP and tPAOP to be weakly (*r* = 0.37) correlated with SI. Other investigators studied volumetric parameters to assess preload in the setting of high interfering pressures [30–32]. Leucke et al. showed intrathoracic blood volume (ITBV) and right ventricular end-diastolic volume (RVEDV) to be good estimates of cardiac preload at high intrathoracic pressures by comparing them with left ventricular end-diastolic volume (LVEDV), the “true cardiac preload,” as measured by rapid acquisition computed tomography [32]. In agreement with these observations, we found GEDVI to be the best preload estimator during IAH, as it correlated best with SI (*r* = 0.52). In our porcine model of IAH, GEDVI first raised from 15.5 ± 1.8 to 17.0 ± 3.1 mL/kg at IAP = 10 mmHg and then decreased towards 14.9 ± 2.7 mL/kg at IAP of 30 mmHg (*P* = 0.07). Our findings are in agreement with data in healthy pigs reported by Quintel et al., showing a significant increase in ITBVI from 18.1 ± 1.6 to 22.4 ± 2.9 mL/kg, when increasing IAP to 13 cmH2O. A further IAP increase to 26 cmH2O reduced ITBVI to 17.9 ± 2.2 mL/kg (*P* = 0.023) [19]. The fact that changes in GEDVI did not reach significance may not surprise since our study was not designed to change intravascular filling status and since SI is determined not only by preload, but also by afterload and cardiac contractility. Importantly, in this study, we did not assess the clinical effect of changing hemodynamics on organ perfusion by measuring lactate or base deficit levels, being another limitation of this study.

## 5. Conclusion

In a porcine model of acute IAH, we found transmission of IAP to the thoracic compartment to be clearly dependent on the stage of the respiratory cycle. Second, IAH results in a decreased respiratory system compliance due to a steep decrease of chest wall compliance. Moreover, GEDVI as well as transmural filling pressures are clearly superior to classical cardiac filling pressures in the assessment of cardiac preload at high intra-abdominal pressures.

## Figures and Tables

**Table 1:** Effect of increasing intra-abdominal pressure (IAP) on pulmonary mechanics, pulmonary function, and global hemodynamics. Pplat plateau airway pressure, PIP peak inspiratory pressure, EVLWI extravascular lung water index, HR heart rate, MAP mean arterial pressure, MPAP mean pulmonary arterial pressure, SI stroke index, SVRI systemic vascular resistance index, and GEDVI global end-diastolic volume index. Data are expressed as mean ± SEM.

IAP (mmHg)	Baseline	10	20	30	Release
*Pulmonary data*					
Pplat (cmH2O)	16 ± 2	17 ± 2	24 ± 2*	32 ± 2*	19 ± 2^∗,†^
PIP (cmH2O)	19 ± 1	20 ± 2	27 ± 2*	34 ± 2*	21 ± 2^†^
EVLWI (mL/kg)	13 ± 2	14 ± 2	15 ± 2	14 ± 3	14 ± 2
paO2/fiO2 (mmHg)	487 ± 13	467 ± 14	487 ± 12	394 ± 15	376 ± 15
paCO2 (mmHg)	39 ± 2	34 ± 2	38 ± 2	38 ± 1	39 ± 1

*Hemodynamic data*					
HR (bpm)	92 ± 5	97 ± 4	92 ± 4	93 ± 4	92 ± 4
MAP (mmHg)	93 ± 4	91 ± 5	98 ± 5	104 ± 5*	98 ± 5
MPAP (mmHg)	26 ± 2	27 ± 2	31 ± 3*	37 ± 5*	34 ± 2^∗,†^
SI (mL/kg)	1.2 ± 0.5	1.3 ± 0.5*	1.2 ± 0.5	1.0 ± 0.5^‡^	1.2 ± 0.5^†^
SVRI (dynes·s/cm^5^·kg)	35 ± 4	28 ± 4*	34 ± 5	41 ± 5^‡^	34 ± 5^†^
GEDVI (mL/kg)	15.5 ± 1.9	17.0 ± 3.1	16.6 ± 3.0	14.9 ± 2.7	17 ± 3.7

For pulmonary data: *P = 0.025 versus baseline, ^†^ *P* = 0.015 versus IAP = 30.

For hemodynamic data: *P = 0.04 versus baseline, ^†^ *P* = 0.05 versus IAP = 30, ^‡^ *P* = 0.05 versus IAP = 10. All data without symbols indicate a *P* value of >0.05.
